# Hematological Changes in the Second Wave of SARS-CoV-2 in North India

**DOI:** 10.7759/cureus.23495

**Published:** 2022-03-25

**Authors:** Akanksha Singh, Shailendra P Verma, Rashmi Kushwaha, Wahid Ali, Himanshu D Reddy, Uma S Singh

**Affiliations:** 1 Pathology, King George's Medical University, Lucknow, IND; 2 Clinical Hematology, King George's Medical University, Lucknow, IND; 3 Internal Medicine, King George's Medical University, Lucknow, IND

**Keywords:** platelet-to-lymphocyte ratio (plr), neutrophil-to-lymphocyte ratio (nlr), second wave of covid-19, sars-cov-2 infection, covid-19 induced cytopathic changes, covid-19 hematological changes, covid-19 india

## Abstract

Background

COVID-19 is a rapidly spreading pandemic caused by SARS-CoV-2. India experienced a second wave peak in mid of April 2021, and it emerged as a medical crisis. This study was taken up to show if the hematological and peripheral blood changes can be used as a readily available tool to demarcate the patients needing ICU care so that the ICU can be utilized more prudently.

Material and method

One hundred reverse transcription-polymerase chain reaction (RT-PCR) confirmed cases of COVID-19, 50 each from ICU and non-ICU wards, were included in this observational study. At the time of admission blood sample was collected for evaluation of hematological parameters.

Results

We noted that 74% of patients admitted in ICU were males and 28% were more than 60 years of age. In ICU patients, the absolute neutrophil count (ANC) was significantly raised when compared to non-ICU cases (p=0.023). The nadir absolute lymphocyte count (ALC) was 0.11x10^9^/L in ICU patients and 0.95x10^9^/L in non-ICU patients. There was a significant increase in neutrophil-lymphocyte ratio (NLR; p<0.001) in ICU patients with a proposed cut-off value of 7.73. Platelet-lymphocyte ratio (PLR) was also raised in ICU patients; however, this increase was not significant (p= 0.623). The proposed cut-off value of PLR is 126.73. A significant reduction in a lymphocyte-monocyte ratio (LMR) was observed in ICU patients when compared to non-ICU cases (p<0.001). Thrombocytopenia was more commonly seen in ICU patients; however, this was not statistically significant. Viral-induced cytopathic effects like plasmacytoid lymphocytes with cytoplasmic granules, the presence of toxic changes in neutrophils, and large-sized platelets were commonly observed in ICU patients.

Conclusion

Our results suggest that hematological parameters like ANC, absolute lymphocyte count (ALC), platelet count, NLR, PLR, and peripheral smear changes are simple assessment factors that can serve as indicators for the severity of COVID-19 and will demarcate the patients who need ICU-care. This will help in the judicious use of ICU facilities for patients who are actually in need.

## Introduction

COVID-19 is caused by a novel coronavirus named severe acute respiratory syndrome coronavirus 2 (SARS-CoV-2), mainly transmitted via droplets and contact surfaces [1]. India experienced a second surge in COVID-19 cases in the middle of March 2021, marking the beginning of the second wave. By the end of the first week of April, the highest number of cases (144,829) was reported [2]. The second wave of COVID-19 nearly collapsed the health care infrastructure. Many hospitals were totally converted to COVID-19 care hospitals, and non-COVID-19 care was almost thinned out. Many other places were converted into makeshift wards to handle this emerging crisis, but people struggled to get a bed in the hospital, and many collapsed gasping for oxygen [2].

This study was done with the aim to show if the hematological and peripheral blood changes can be used as a tool to demarcate the patients who will need intensive care unit (ICU) so that the ICU care can be utilized more prudently, especially when we were anticipating a third wave soon. 

COVID-19 mainly targets the patient's lungs but can damage other organs as well. Most of the patients present with lower respiratory infections like fever, cough, sore throat, myalgia, dyspnoea. However, a few patients progress to acute respiratory distress syndrome [1,3].

Viral infections such as infectious mononucleosis, viral hepatitis, cytomegalovirus infections, human immunodeficiency virus infections (HIV) are known to change the cell numbers and morphology in the peripheral blood smear. In addition to numerical changes like leukocytosis, leukopenia, neutrophilia, neutropenia, monocytosis, monocytopenia, lymphocytosis, lymphopenia, and thrombocytopenia, they can also change the morphology of the cells. This may be in the form of the presence of atypical lymphocytes, inclusion bodies, vacuolization, and blast-like cells. Such hematological changes are also observed in COVID-19 patients [3-5].

## Materials and methods

This descriptive study was carried out after obtaining institutional ethical clearance. A total of 100 reverse transcription-polymerase chain reaction (RT-PCR) positive cases, 50 from the intensive care unit (ICU) and 50 from non-ICU COVID-19 wards, were enrolled in this study conducted at a tertiary care center in north India (Department of Pathology, King George's Medical University, Lucknow). This study was done from May to June 2021 and was approved by the institutional ethical clearance committee. 

Basic clinical details and demographic assessment was done. Blood samples were collected under aseptic conditions at the time of admission in ICU and non-ICU COVID-19 wards. To analyze hematological parameters, 2 ml of blood was collected in an ethylenediaminetetraacetic acid vacutainer. A peripheral blood smear was made with fresh blood and stained with Leishman's stain. Complete blood count (CBC) was done using the flow cytometric technique in the Sysmex XN-500 instrument (Sysmex Corporation, Kobe, Japan).

Various parameters were compared between ICU and non-ICU cases using independent sample t-tests. Pearson's correlation and binary multivariate logistic regression were studied between all parameters. IBM-SPSS statistics software (version 24.0; IBM Inc., Armonk, USA) was used for data analysis.

## Results

The demographic profile of patients admitted in ICU and non-ICU groups are mentioned in Table 1. The mean age of patients admitted in ICU was significantly higher (54.40± 16.89 years vs. 43.08± 14.66 years) than in the non-ICU group. Twenty-eight percent of patients admitted in ICU were more than 60 years of age. In contrast, only 8% of patients were more than 60 years in non-ICU cases. This indicates that in the second wave of SARS-CoV-2 older people were at high risk of developing more severe infection and requiring ICU care. Seventy-four percent of patients admitted to ICU were males. No such difference was found in non-ICU cases (see Table 1).

**Table 1:** Demographic profile of patients * statistically significant

Variables	ICU cases			Non-ICU cases			Chi-square	P-value
	n=50	%	Mean ±SD	n=50	%	Mean ±SD		
Sex								
Male	37	74.00		22	44.00		8.10	0.004^*^
Female	13	26.00		28	56.00			
Age in years			54.40±16.89			43.08±14.66	15.65	0.001
<25	2	4.00		8	16.00			
25-60	34	68.00		38	76.00			
>60	14	28.00		4	8.00			

ICU patients had associated comorbidities like diabetes (five patients), hypertension (12 patients), cardiovascular diseases (three patients), respiratory diseases (four patients), and obesity (22 patients).

COVID-19 infection was associated with leukocytosis. The mean leukocyte count was 15.464±14.572X10^9^/L and 12.682±14.786x10^9^/L in ICU and non-ICU cases, respectively (see Table 2).

**Table 2:** Hematological parameters of the study cohorts Hb - hemoglobin; TLC - total leucocyte count; MPV - mean platelet volume; MCV - mean corpuscular volume; MCH - mean corpuscular hemoglobin; MCHC - mean corpuscular hemoglobin concentration; RDW - red cell distribution width; HCT - hematocrit; RBC - red blood cell; ANC - absolute neutrophil count; AMC - absolute monocyte count; ALC - absolute lymphocyte count * statistically significant

Parameters	ICU group (n=50)		Non-ICU group (n=50)		p-value
	mean	±SD	mean	±SD	
Hb (gm/dl)	11.57	2.49	11.21	2.79	0.498
TLC (x10 ^9^/L)	15.464	14.572	12.682	14.786	0.346
Neutrophils (%)	84.92	7.31	77.90	9.21	<0.001^*^
Lymphocytes (%)	10.58	7.15	17.12	8.34	<0.001^*^
Eosinophil (%)	2.72	0.88	3.60	1.40	<0.001^*^
Monocytes (%)	1.80	0.73	1.38	0.49	0.0011^*^
Basophil (%)	0.00	0.00	0.00	0.00	0.062
ANC (x10 ^9^/L)	12.95	13.01	8.507	3.825	0.023^*^
ALC (x10 ^9^/L)	1.391	1.401	1.708	0.991	0.194
AMC (x10 ^9^/L)	0.269	0.216	0.145	0.091	<0.001^*^
Neutrophil lymphocyte ratio (NLR)	12.07	7.78	5.81	2.80	<0.001^*^
Platelet lymphocyte ratio (PLR)	168.59	128.08	155.48	136.27	0.623
Lymphocyte monocyte ratio (LMR)	7.59	6.58	13.03	6.05	<0.001*
Platelet count (x10 ^9^/L)	166	115	208	105	0.112
MPV (fl)	9.97	1.16	9.56	1.39	0.994
Total RBC (x10 ^12^/L)	4.07	0.97	4.07	0.91	0.032
MCV (fL)	84.59	10.42	78.96	15.09	0.058
MCH (pg)	28.73	3.39	27.31	4.03	0.567
MCHC (g/dl)	34.02	1.73	33.85	1.30	0.578
RDW (%)	17.90	2.32	18.18	2.78	0.402
HCT (%)	34.24	8.26	32.86	8.09	0.498

The mean neutrophils and monocytes were significantly higher, and the mean lymphocytes and eosinophils were significantly lower in ICU patients. In the ICU group, lymphopenia with absolute lymphocyte count (ALC) <1x10^9^/L was observed in 23 patients, with 18 having moderate lymphopenia (ALC: 0.5-1x10^9^/L) and five having severe lymphopenia (<0.5x10^9^/L). The nadir absolute lymphocyte count (ALC) was 0.112x10^9^/L in ICU patients and 0.95x10^9^/L in non-ICU patients. The mean absolute neutrophil count (ANC) was 84.92x10^9^/L in ICU cases compared to 77.90x10^9^/L in non-ICU cases. An increase in the neutrophil count was more in ICU cases than in non-ICU cases.

There was no significant difference in hemoglobin, mean platelet count, mean platelet volume (MPV), red blood count (RBC), mean corpuscular hemoglobin (MCH), mean corpuscular hemoglobin concentration (MCHC), red cell distribution width (RDW), and hematocrit (HCT) between ICU and non-ICU cases. The mean corpuscular volume of red blood cells (MCV) was in the normal range in both ICU and non-ICU cases (see Table 2).

In the ICU group, 15 patients had mild thrombocytopenia (platelet count 100-150x10^9^/L), 13 patients had moderate thrombocytopenia (platelet count 50-100x10^9^/L), and only two patients had severe thrombocytopenia (platelet count <50x10^9^/L). Among non-ICU patients, only 12 patients had thrombocytopenia (four had mild and eight had moderate thrombocytopenia), while none of the non-ICU patients were severely thrombocytopenic.

Table 3 shows the correlations of patients in ICU and their hematological parameters. Pearson's correlation coefficient of neutrophils and monocytes were positively correlated, whereas lymphocytes and eosinophils were negatively correlated with ICU cases.

**Table 3:** Correlations of hematological parameters and ICU admission Hb - hemoglobin; TLC - total leucocyte count; RBC - red blood count; MCV - mean corpuscular volume; MCH - mean corpuscular hemoglobin; MCHC - mean corpuscular hemoglobin concentration; RDW - red cell distribution width; HCT - hematocrit * statistically significant

Parameters	Pearson's correlation coefficient	p-value
Hb	0.069	0.498
TLC	0.095	0.346
Neutrophils	0.392	<0.001^*^
Lymphocytes	-0.391	<0.001^*^
Eosinophil	-0.355	<0.001^*^
Monocytes	0.323	0.001^*^
Platelets	-0.187	0.062
MPV	0.160	0.112
Total RBC	0.001	0.994
MCV	0.214	0.032^*^
MCH	0.190	0.058
MCHC	0.058	0.567
RDW	-0.056	0.578
HCT	0.085	0.402

**Figure 1:**
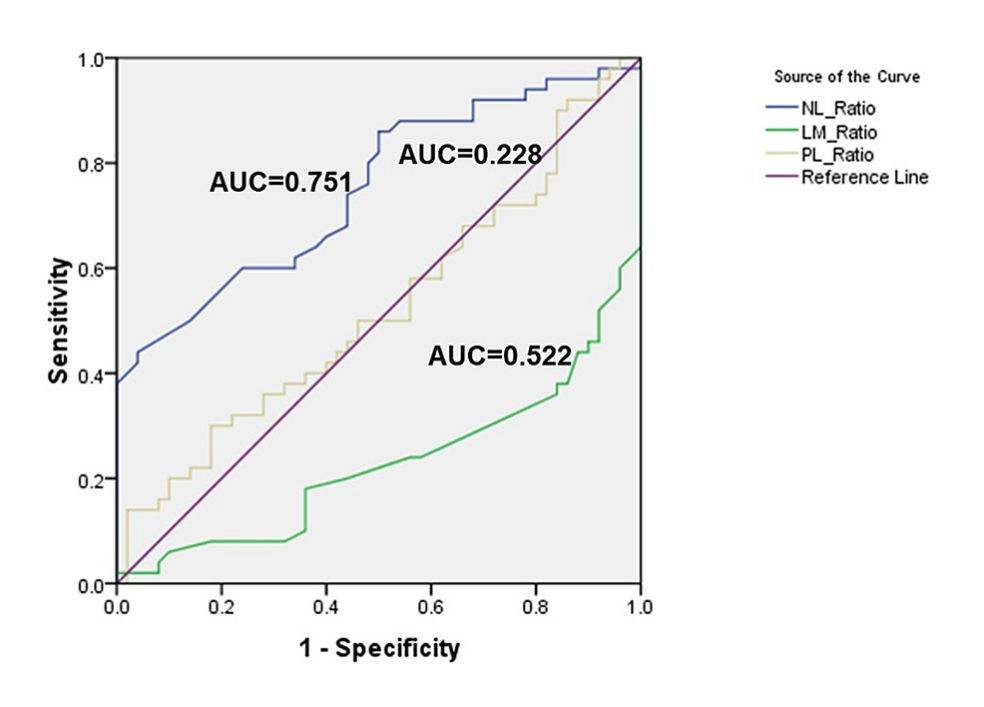
Receiver operating characteristic (ROC) curve The receiver operating characteristic (ROC) curve shows the relative diagnostic performance of neutrophil-lymphocyte ratio (NLR), lymphocyte-monocyte ratio (LMR), and platelet-lymphocyte ratio (PLR). The area under the curve (AUC) of LMR is less than 0.50; hence it can not be used as a potential diagnostic marker. The cut-off values detected are 7.73 for NLR and 126.73 for PLR.

There are no unified laboratory cut-off values for neutrophil-lymphocyte ratio (NLR), lymphocyte monocyte ratio (LMR), and platelet lymphocyte ratio (PLR). Therefore, we analyzed the optimum cut-off values by receiver operating characteristic (ROC) curve analysis (Figure 1). The areas under the curve (AUC) of NLR, LMR, and PLR were 0.751, 0.228, and 0.522, respectively (Table 4). LMR cannot be used as a potential diagnostic biomarker as its AUC is less than 0.50. The optimal cut-off values were 7.73, 10.0, and 126.73 for NLR, LMR, and PLR. NLR was significantly raised in ICU cases. PLR was raised in ICU cases when compared with non-ICU cases; however, statistically, this was not significant. LMR was significantly raised in non-ICU cases (Table 2).

**Table 4:** Receiver operating characteristic analysis of various ratios proposed for categorization of ICU or non-ICU patients

Ratio	Area	Standard error	p-value	95% confidence interval		Cut-off value
				Lower bound	Upper bound	
Neutrophil-lymphocyte ratio (NLR)	0.751	0.05	<0.001	0.656	0.85	7.73
Lymphocyte-monocyte ratio (LMR)	0.228	0.05	<0.001	0.134	0.32	10.0
Platelet-lymphocyte ratio (PLR)	0.522	0.06	0.700	0.408	0.64	126.73

Peripheral smear changes in COVID-19 infections

A peripheral blood smear was examined carefully in all ICU and non-ICU cases (see Table 5).

**Table 5:** Peripheral smear morphological changes in ICU and non-ICU cases

Morphology of cells	ICU cases (n=50)	Non-ICU cases (n=50)
Lymphocytes		
Plasmacytoid cells	44	30
Cytoplasmic pseudopods	40	20
Cytoplasmic granules	28	22
Prominent nucleoli	36	21
Neutrophils		
Coarse cytoplasmic granules	30	10
Cytoplasmic vacuoles	27	12
Fetus shaped nucleus	12	2
Pseudo-pelger cells	25	8
Elongated nucleoplasm	20	4
Monocytes		
Cytoplasmic vacuoles	38	12
Cytoplasmic granules	12	4
Platelets		
Large giant pleomorphic forms	42	20

**Figure 2:**
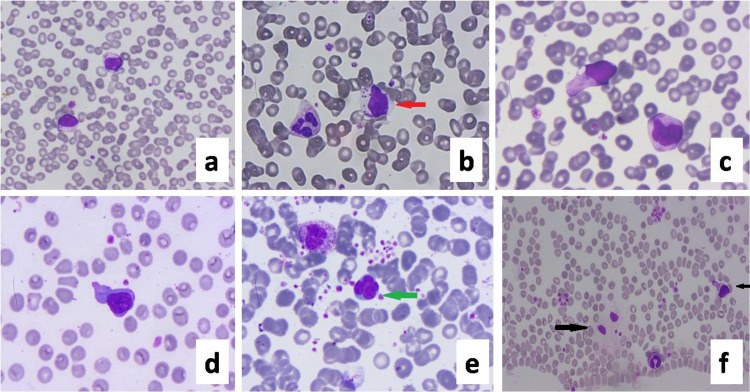
Peripheral blood smear showing various changes in lymphocyte a: atypical lymphocytes showing round to irregular nucleus, opened chromatin, prominent nucleoli, abundant cytoplasm with distinct variable azurophilic granules (virocyte/covicyte); b: large granular lymphocyte (red arrowhead); c and d: Downey cells showing irregular nucleus, hyperbasophilic cytoplasm with pod formation; e: apoptotic lymphocyte (green arrowhead); f: macrophage in circulation (broad black arrowhead) adjacent to atypical lymphocyte (short black arrowhead). Leishman 400-1000x

Lymphocytes had large azurophilic granules in the cytoplasm (large granular lymphocytes). The nucleus was round to indented with coarse nuclear chromatin and occasional prominent nucleoli. Abundant basophilic cytoplasm with cytoplasmic pseudopod formation was a frequent finding. Some atypical lymphocytes with plasmacytoid morphology, eccentric nuclei, perinuclear hoff were also seen (Figure 2). Many apoptotic bodies were noted. These findings were found in most ICU patients. The percentage of atypical lymphocytes also correlated significantly.

**Figure 3:**
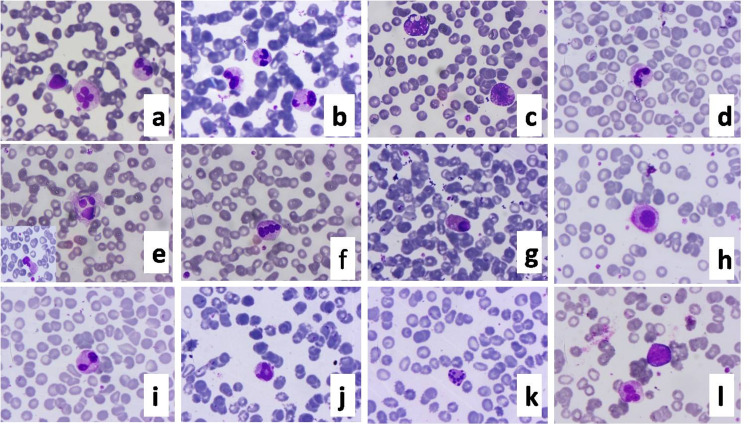
Peripheral blood smear showing various changes in neutrophils/granulocytes a and b: dysplastic neutrophils; c: toxic changes like coarse cytoplasmic granules, vacuoles, and irregular nuclear lobulations; d: C-shaped, fetus-like COVID-19 nuclei; e: granulocytes showing cell rupture/lysis; f: dysplastic cell showing nuclear clumping and rounding of the nucleus; g: eosinophil undergoing pyknosis; h: karyolysis, melting of nuclear chromatin with enzymes released by the lysosomes of the dead cells, hypergranular cytoplasm with perinuclear hypogranular areas; i: dysplastic cell showing pseudo-Pelger like nuclei; j: dysplastic change in the form of ring nucleus; k: apoptotic cells; l: circulating myelocyte. Leishman 200-400x

Neutrophilia was more in ICU patients. Neutrophils showed toxic changes in the form of coarse cytoplasmic granules, the presence of cytoplasmic vacuoles. C-shaped fetus-like nuclei with numerous nuclear projections were present. Nuclear detachments with elongated nucleoplasm, pseudo-Pelger Huet cells were seen (Figure 3).

**Figure 4:**
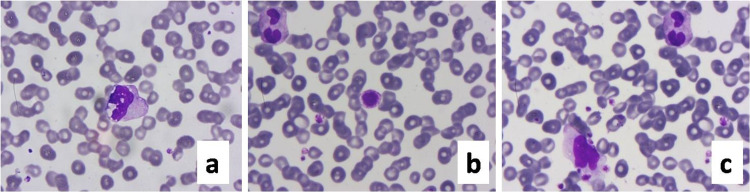
Peripheral blood smear a: activated monocyte with multiple cytoplasmic vacuoles and fine granules; b: circulating giant platelet with irregular cytoplasm; c: activated lymphocyte with adherent platelets. Leishman 400-1000x

Activated monocytes with cytoplasmic vacuoles and fine granules were present (Figure 4a). Thrombocytopenia, large giant pleomorphic platelets, and platelets clumping to activated lymphocytes were seen commonly in ICU cases (Figure 4b). Pyknosis (shrinkage of the nucleus), karyorrhexis (rupture of nuclear membrane), karyolysis (melting of nuclear chromatin with enzymes) were frequent findings in ICU patients.

## Discussion

We report hematological changes in 100 patients, 50 each from ICU and non-ICU, who were RT-PCR confirmed COVID-19 cases. This study aimed to determine the magnitude of hematological changes in the COVID-19 second wave and to show how significantly they were altered in ICU cases. Simple and relatively inexpensive tests like CBC and peripheral smear evaluation can help in patients' risk stratification. 

The literature search shows limited reviews on the causes of these hematological changes. Many researchers have claimed that neutrophilia heralds the beginning of cytokine storm and hyperinflammatory response [6,7]. Morphological abnormalities in the form of nuclear changes, karyorrhexis, pyknosis, and karyolysis have been demonstrated in circulating neutrophils of patients having more severe disease and secondary complications requiring ICU care. This usually precedes lymphocyte-related changes [7].

Lymphopenia was a common finding in our patients. More severe lymphopenia was observed in ICU patients. Those requiring ICU had a much lower ALC, the median nadir of ALC of 0.112x10^9^/L in ICU patients compared to 0.95x10^9^/L in non-ICU cases. This may be associated with the defective immune response to the virus. Similar findings were also seen in the first wave of SARS-CoV-2 by Fan et al. [8]. Severe lymphopenia in patients needing ICU care can be explained by lymph node destruction, suppression of lymphocytes during lactic acidosis, and binding of SARS-CoV-2 to angiotensin-converting enzyme two receptors on lymphocytes [9]. In Huang et al. study including 140 patients reported lymphopenia in 63% and leucopenia in 25% of patients. Huang et al. reported that significant predictors of ICU admission are leukocytosis (2.0-fold increased in ICU patients), neutrophilia (4.4-fold increased), and lymphopenia (0.4-fold, i.e., decreased) [10].

Wang et al. in their study of 138 patients, reported that patients with a higher white blood cell count (1.5-fold), higher neutrophil count (1.7-fold), and lower lymphocyte count (0.9-fold) are more likely to need ICU care [11].

Qin et al. studied various haematological parameters in 452 patients and found that severe cases had higher leukocyte (5.6 vs. 4.9x10^9^; p<0.001) and neutrophil (4.3 vs. 3.2x10^9^; p<0.001) counts, lower lymphocytes counts (0.8 vs.1.0x10^9^; p<0.001), a higher neutrophil-to-lymphocyte ratio (5.5 vs. 3.2; p<0.001), as well as lower percentages of monocytes (6.6 vs. 8.4 %; p<0.001), eosinophils (0.0 vs. 0.2%; p<0.001), and basophils (0.1 vs. 0.2%; p=0.015) when compared to non-severe cases [12]. An increased NLR is suggestive of severe infection and need of ICU care [13]. Our study showed a significant increase in NLR in ICU cases.

Literature search shows few articles that have used NLR, LMR, and PLR to assess disease severity. In our study, cut-off points were observed for NLR, LMR, and PLR using ROC curve analysis. We propose a cut-off value of 7.73 and 126.73 for NLR and PLR. LMR cannot be used as a potential diagnostic biomarker as its AUC is less than 0.50. Citu et al. proposed a cut-off value of 9.1 for NLR [14].

There is significant reduction in CD45+, CD3+, CD4+, CD8+, CD19+and CD16/56+ count. However, CD4 to CD8 ratio is not reversed. T-helper-1 (Th1) function is activated due to the increased concentration of inflammatory mediators such as IL-1B, IL-6, IL-12, IL-18, IL-33, CXCL10, CCL2, and tumor necrosis factor-alpha (TNF-alpha) [15].

SARS-CoV-2 inhibits bone marrow hematopoiesis through specific receptors causing lymphopenia and thrombocytopenia [16]. It also causes platelet destruction by antiplatelet autoantibodies. Platelet production is also impaired due to direct viral insult to the bone marrow and impaired fragmentation of megakaryocytes due to COVID-19 induced damage to the lung and pulmonary capillary bed [15]. However, data on thrombocytopenia is variable in various studies. Few studies have shown the incidence of thrombocytopenia as high as 57% amongst non-survivors [9]. However, platelet count below 100x10^9^/L occurs only in 5% of hospitalized cases [17]. At our center, severe thrombocytopenia (platelet count <50x10^9^/L) was seen in two ICU cases and no patients in the non-ICU group. A meta-analysis performed on peer-reviewed data by Lippi et al. projected that significant thrombocytopenia is associated with severe SARS-CoV-2 infection [6]. The literature search also shows the association of thrombocytopenia with severe acute respiratory syndrome (SARS) outbreaks in the past [18-20]. In association with other hematological parameters, thrombocytopenia is a strong predictor of ICU admission.

Secondary hemophagocytic lymphohistiocytosis has been found in many patients with SARS-CoV-2 infection. This infection causes activation and proliferation of macrophages and a surge in cytokine levels leading to multiorgan failure and mortality [21].

There are several limitations of this study. First, the power of the study could not be assessed due to the limited sample size. Second, this was a single-center study and not from multiple centers. Third, the final medical outcome was not part of our study; hence, it was not included. We also admit that correlation of onset of symptoms and days of illness with hematological parameters is important, which was not done in our study as we included only the first collected sample at the time of admission. 

## Conclusions

The literature search shows limited studies on the association of hematological variations and morphological changes in blood cells with the severity of SARS-CoV-2 during the second wave of the pandemic in Northern India. Our study revealed that patients in ICU had higher ANC, deeper nadir ALC, higher NLR, higher PLR, lower LMR, and severe thrombocytopenia. Virus-induced cytopathic changes were more common in ICU patients. We propose a cut-off value of 7.73 and 126.73 for NLR and PLR for COVID-19 patients needing ICU care. Careful assessment of hematological parameters and meticulous examination of peripheral blood smear can be used for risk stratification of patients. It can act as a tool to identify patients who need ICU care.
